# A decision support system to determine optimal ventilator settings

**DOI:** 10.1186/1472-6947-14-3

**Published:** 2014-01-10

**Authors:** Fatma Patlar Akbulut, Erkan Akkur, Aydin Akan, B Siddik Yarman

**Affiliations:** 1 Department of Computer Engineering, Istanbul Kültür University, Istanbul, Turkey; 2 Directorate General of Health for Border and Coastal Areas, Republic of Turkey, Ministry of Health, Istanbul, Turkey; 3 Department of Electrical and Electronics Engineering, Istanbul University, Istanbul, Turkey

**Keywords:** Ventilator settings, Decision support systems, Artificial neural networks, Bayesian model

## Abstract

**Background:**

Choosing the correct ventilator settings for the treatment of patients with respiratory tract disease is quite an important issue. Since the task of specifying the parameters of ventilation equipment is entirely carried out by a physician, physician’s knowledge and experience in the selection of these settings has a direct effect on the accuracy of his/her decisions. Nowadays, decision support systems have been used for these kinds of operations to eliminate errors. Our goal is to minimize errors in ventilation therapy and prevent deaths caused by incorrect configuration of ventilation devices. The proposed system is designed to assist less experienced physicians working in the facilities without having lung mechanics like cottage hospitals.

**Methods:**

This article describes a decision support system proposing the ventilator settings required to be applied in the treatment according to the patients’ physiological information. The proposed model has been designed to minimize the possibility of making a mistake and to encourage more efficient use of time in support of the decision making process while the physicians make critical decisions about the patient. Artificial Neural Network (ANN) is implemented in order to calculate frequency, tidal volume, *FiO*_2_ outputs, and this classification model has been used for estimation of pressure support / volume support outputs. For the obtainment of the highest performance in both models, different configurations have been tried. Various tests have been realized for training methods, and a number of hidden layers mostly affect factors regarding the performance of ANNs.

**Results:**

The physiological information of 158 respiratory patients over the age of 60 and were treated in three different hospitals between the years 2010 and 2012 has been used in the training and testing of the system. The diagnosed disease, core body temperature, pulse, arterial systolic pressure, diastolic blood pressure, PEEP, *PSO*_2_, pH, *pCO*_2_, bicarbonate data as well as the frequency, tidal volume, *FiO*_2_, and pressure support / volume support values suitable for use in the ventilator device have been recommended to the physicians with an accuracy of 98,44%. Performed experiments show that sequential order weight/bias training was found to be the most ideal ANN learning algorithm for regression model and Bayesian regulation backpropagation was found to be the most ideal ANN learning algorithm for classification models.

**Conclusions:**

This article aims at making independent of the choice of parameters from physicians in the ventilator treatment of respiratory tract patients with proposed decision support system. The rate of accuracy in prediction of systems increases with the use of data of more patients in training. Therefore, non-physician operators can use systems in determination of ventilator settings in case of emergencies.

## Background

The use of decision support systems [1] in health provides improvement in terms of the quality of health and care, early diagnosis of diseases, prevention of person related errors, lowering costs and providing the patients with the optimum treatment.

Today, most physicians prefer the decision support systems in the interest of the effective operation and early determination of the most appropriate option in order to avoid the growing problem related to the management of medical information. The decision support systems are the most ideal aids in either detection or treatment of a disease, or determination of the most appropriate drug.

A basic requirement of our survival is to take a sufficient amount of air and to transfer it to all our cells. While heart and blood cells are responsible for distribution, our lungs perform the exchange of oxygen (*O*_2_) and carbon dioxide (*CO*_2_) by holding a medietary position between the atmosphere and the internal organs. The delivery of oxygen and removal of carbon dioxide, which are the two most important components of the inspiration - expiration process is, called “breathing” [2]. The respiratory function is autonomously carried out in healthy living beings and is artificially carried out in those with lung disease and respiratory failure. The device that is used in this process is called as the ventilator device [3] and the process is called ventilation.

The physiological parameters determined in accordance with the findings of the patient who is connected to the ventilator device change simultaneously depending on the instantaneous status of the patient after the device has been inserted. The most critical point here is to determine the device parameters in the desired accuracy. Since the task of specifying the parameters of ventilation equipment is carried out entirely by a physician, the physician’s knowledge and experience in the selection of these settings have a direct effect on the accuracy of his/her decisions. The process of determination of the device parameters with a decision support system to be developed for this decision process can be completed with minimum error by predicating the instantaneous status of the patient. By this way, the decision making processes of physicians can be implemented in a faster and a more accurate manner.

This study describes a decision support system designed to automatically determine the ventilator settings in consideration of the ventilation data obtained from two different hospitals. For the model system in our study, COPD (Chronic Obstructive Pulmonary Disease) and CVD (Cerebrovascular Disease) have been used as respiratory diseases. COPD [4] is a disease that causes the obstruction of the bronchi on the long term and that has no treatment. ARDS [5] is the acute respiratory distress syndrome caused by the increase in the alveolar capillary permeability. CVD [6] is a group of disorders with symptoms related to the damaged brain region associated with the blockage or bleeding of the arteries supplying the brain. Although CVD is not a respiratory disease, almost all CVD patients are treated with ventilation devices.

According to the Turkish Statistical Institute’s report of causes of death statistics [7], 16.311 patients died of disease caused by COPD (J40-J44) and 30.103 patients died of disease caused by CVD (I60-I69) in 2009. In total mortality of 2009, it corresponds to 16,5%.

For this reason, the management of mechanical ventilation devices used in intensive care units is a critical process. Our goal is to minimize errors in this process and prevent deaths caused by incorrect configuration of ventilation devices. This system is designed for the facilities without having lung mechanics like cottage hospitals in country regions. Metropolitan cities have fully equipped hospitals with experienced medical personnel, but on the other hand less experienced doctors can benefit from this system. Our proposed method is facilitated upon making the medical data of patients meaningful with help of an Artificial Neural Network (ANN) [8], and the determination of the preferred treatment factors.

Below, further studies conducted so far with regards to the subject of our study have been summarized.

In the study of Adeney, Ennett, Frize and Korenberg [9], this methodology is used and a model using two-stage ANN is presented. It is observed from the test results that a high success rate is provided with regards to the estimation of the parameters.

A similar study has simultaneously performed the inspiration – expiration of the intensive care patients by a ventilator device [10]. The model working in accordance with the data such as the patient’s breathing frequency, the density of oxygen in their blood and the flow wavelength of the blood has estimated the ideal working time.

Borrello’s study [11] is a detailed modeling of a prototype which is thought as a replacement for the standard ventilator devices and which provides assistance for the operator with regards to the parameters to be selected through its own processor and sensor.

In the study of Tehrani [12], a closed-loop decision support system that foresees the leaving time of the patient from the ventilator depending on values *PCO*_2_ (Partial Pressure of Carbon Dioxide), *PSO*_2_ (Partial Pressure of Sulfur Dioxide), *FiO*_2_ (Fraction of Inspired Oxygen), PEEP (Positive End-Expiratory Pressure) parameters was proposed.

In another study [13], simulations have been implemented in the MATLAB environment by using the hybrid algorithm for the ventilators used in intensive care units. With the designed model, the settings regarding the parameters of the blood gasses like *FiO*_2_, PEEP, Pinsp, Vrate are recommended to the physician by the system.

Another computer aided ventilator prototype study [14] was developed by Ahmedi and Bates. Prototype ventilator opens and closes the valve by the help of the computer program, which sustains the process of inspiration and expiration.

Zhu [15] and Allerød [16] proposed ventilation settings with a decision support system according to the patient’s physiological characteristics of the patient to be undergone ventilation, by using ANNs. While Zhu has used the data from 28 ARDS patients in his study; Allerød used the data from 20 CABG patients. In both studies, it is shown that patient characteristics are important in the selection of the ventilator settings.

As a result, it is seen in the research articles [17] related to the methodology of the ventilator devices controlled by the smart control techniques that the influence of the decision support systems on the decisions of the physician have been quite successful.

Er, Yumusak and Temurtas [18] presented a comparative chest diagnosis; for chronic obstructive pulmonary, pneumonia, asthma, tuberculosis and lung cancer diseases which was realized by using multilayer, probabilistic, learning vector optimization, and generalized regression.

Gil et al. [19] used ANN models as tools for support in the medical diagnosis of urological dysfunctions. They developed two types of unsupervised and one supervised neural network to distinguish and classify between ill and healthy patients.

Ushida et al. [20] used fuzzy neural network analysis (FNN) of health check-up data to provide a personalized novel diagnostic and therapeutic method involving the y-GTP level and the WBC count. They performed a logistic regression analysis, including adjustment to ensure FNN analysis was statistically reliable.

Heckerling et al. [21] used ANN coupled with genetic algorithms to evolve combinations of clinical variables optimized for predicting urinary tract infection for women. Their method revealed that parsimonious variable sets accurate for predicting urinary tract infection, and novel relationships between symptoms, urinalysis findings, and infection.

The Faculty of Medicine, the Electronic and Computing Engineering Department of the Federal University of Rio de Janeiro (UFRJ) and the Electrical Engineering Department of the Federal Center of Technological Education (CEFET-RJ) worked on a collaborative research project to develop a decision support system for smear negative pulmonary tuberculosis (SNPT) [22]. This project aims to develop, through a multi-disciplinary, multi-institutional, innovative and cost-effective approach, new paradigms to prevent the disease progression and support the rapid evaluation of new therapies.

Moein, Monadjemia and Moallem [23], investigated typical disease diagnoses using ANNs. After selecting some symptoms of eight different diseases, MLP neural network was used with a fuzzy approach to get more accurate results.

Kamruzzaman and Islam [24], proposed an algorithm, called rule extraction from ANNs (REANN), to extract rules from trained ANNs for medical diagnosis conditions like breast cancer, diabetes and lenses problem.

Lisboa and Taktak [25] conducted to assess the benefit of ANNs as decision making tools in the field of cancer. Their study describes the work being done in this area over the last decade.

Clinical applications of ANNs provide benefits in many areas, and there are still many more perspectives to be examined like ethics and clinical prospects [26].

In the Methods Section, the methodology and design elements of the decision support system are detailed. Experimental results and conclusion are given in Section “Results and discussion” and Section “Conclusions”, respectively.

## Methods

### Data collection and analysis

A medium-sized data set has been used in order to test the accuracy of the model. Data set includes information of 158 respiratory patients who were over the age of 60 and were collected from 3 different hospitals between the years 2010 and 2012.These hospitals are Kartal Kosuyolu Training and Research Heart Hospital, Taksim Training and Research Hospital and Marmara University Pendik Training and Research Hospital. We are allowed to collect patients’ data for this research from Health Directorate of Istanbul and hospital administrations respectively. All data is taken from patient registration forms of intensive care units from hospital archives. While collecting data set, 1 day data of each patient was considered and the overall value was calculated and recorded as the data of the concerned person. Because deviations of the measurements made during the day were not remarkable, we decided to work with the daily averaged values. Data is used as untouched, no statistical analysis or normalization was applied to collected data.

There are 15 different physiological parameters for each patient in the data set. Each parameter has been considered as a different feature. Parameters of the data set were determined as follows: core body temperature, pulse, arterial systolic pressure, diastolic blood pressure, PEEP, *pSO*_2_, pH, *pO*_2_, *pCO*_2_, bicarbonate, frequency as inputs, *FiO*_2_, tidal volume, pressure support and volume support as outputs. Because of being the intensive care units of the hospitals are too busy, doctors only record the important parameters of ventilation devices of their patients. Therefore, it is not possible to find the same parameters on the patient registration forms. This is why we have chosen the values of frequency, tidal volume, *FiO*_2_ and ventilation modes as output parameters. There are three types of ventilation modes used in the system; SIMV (Synchronized Intermittent Mandatory Ventilation) as pressure support mode and CPAP’s (Continuous Positive Airway Pressure) both volume support and pressure support modes.

### Proposed model

**Figure 1:**
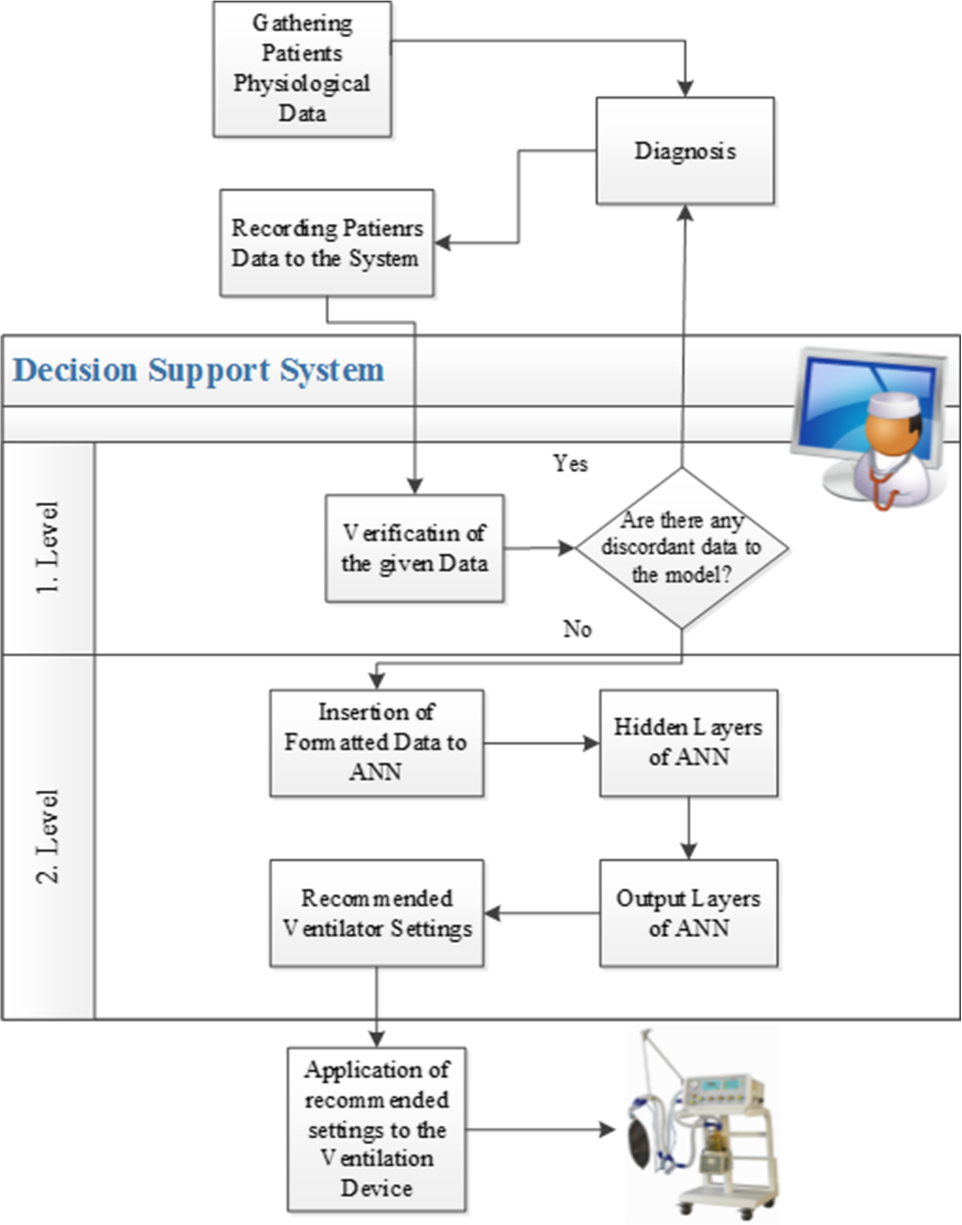
Flow diagram of proposed model.

The decision support system proposed consists of two stages. The first stage is estimating the disease by using previous years’ patient data with Bayes and the second stage is estimating the ventilator settings using ANN. The system architecture and methodology of operation are shown in Figure 1.

If we look at the detailing of the proposed system, the first stage is the part that confirms the physiological values of the patients. At this level, all findings are evaluated by the system, and are checked prior to entering to ANN. The diagnosis regarding the disease of the patient is controlled by the available data in the system. Bayesian model used in the determination of the disease is described in disease detection by Bayesian forecast model section. The system, after checking the entries, evaluates the data that is inappropriate for to the model as a possible faulty entry and alerts the physician. After the physician confirms the data, second stage is put into operation. We do not prefer to feed data directly to ANN, because sub setting the whole data with Bayesian model gives more accurate results in ANN experiments. Only determined disease oriented data is used in ANN. In this point of view, first stage of the system acts like the supervisor of the second stage.

In the second stage of the system, the data related to the patient is formed and entered to ANN. By using the fore mentioned data, ANN produces the tidal volume, frequency and *FiO*_2_ ventilator settings to be used in treatment of patient. The treatment starts when the physician installs the proposed settings to the ventilator. Operation details of ANN are presented in Calculation of the ventilator settings with Artificial Neural Network.

### Disease detection by Bayesian forecast model

Bayesian [27] approach commonly used in the decision support systems is based on an objective point of view in a probabilistic examination of knowledge. The approach focuses on the multiple stages of the information, rather than its accuracy, and allows for the calculation of the future probabilities.

During the modelling of the system, D is considered as the disease and *x*_*n*_ as the findings of disease; which are shown in the equation 1 below.

(1) $$D=(x_{1}⋂D)⋃(x_{2}⋂D)⋃\cdots ⋃(x_{n}⋂D)$$

**Figure 2:**
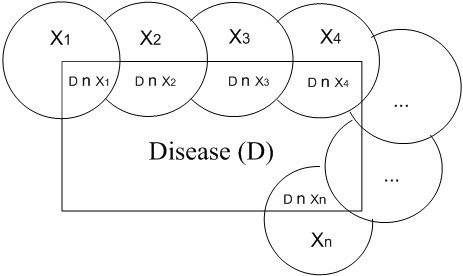
Disease and physiologic parameters.

Representation of the intersection of disease and findings is shown in Figure 2, all findings are considered independently.

P(D) and the possibility of any disease is given in Equation 2.

(2) $$\begin{array}{ll} P(D) & =P\left[\left(x_{1}⋂D)⋃(x_{2}⋂D)⋃\cdots ⋃(x_{n}⋂D\right)\right]\\ & =\overset{n}{\underset{i=1}{\sum }}P(x_{i}⋂D)=\overset{n}{\underset{i=1}{\sum }}P(x_{i})P(D\setminus x_{i}) \end{array}$$

The conditional probability notation *P*(*D*|*x*_*n*_) has been used for expressing a random D event based on a different random x parameter. The conditional probability of P(D) disease according to the known *x*_*n*_ parameter is given in Equation 3.

(3) $$P(D|x_{n})=\frac{P(D⋂x_{n})}{P(x_{n})}$$

Weight of the input parameters (4) within the model is calculated by dividing the number of persons corresponding to the physiological parameters into the total number of persons, when calculating the disease probabilities.

(4) $$w_{n}=\frac{x_{n}}{\overset{n}{\underset{i=0}{\sum }}x_{n}}$$

(5) $$F=w_{1}x_{1}+w_{2}x_{2}+\cdots +w_{n}x_{n}$$

Distribution is directly related to the number of persons corresponding to the physiological parameters. Total number of patients is the normalizing factor which is used to guarantee the sum of all probabilities is equals to 1.

Disease forecast model, which is the first step of the system design, has been used for the data of 158 different patients; the physiological parameters and predicted probabilities of diseases were given in Table 1 through equation 5. As seen in Table 1, the probabilities of COPD, ARDS and CVD were predicted with the accuracies of 63%, 66% and 73%.

**Table 1:** Probabilities of occurrence of COPD, ARDS and CVD diseases

		**Physiological parameter (x)**	**COPD (*F***_***COPD***_**)**	**ARDS (*F***_***ARDS***_**)**	**CVD (*F***_***CVD***_**)**
Symptom	1	Core body temperature	0,267	0,738	0,825
	2	Pulse	0,167	0,631	0,873
	3	Arterial systolic pressure	0,633	0,200	0,413
	4	Diastolic blood pressure	0,367	0,354	0,206
Respiratory	5	FiO2	0,933	0,769	0,778
	6	Frequency	0,567	0,600	0,905
	7	Tidal volume	0,700	0,754	0,683
	8	PEEP	0,600	0,662	0,683
Blood gas	9	pSO2	0,700	0,738	0,857
	10	pH	0,767	0,615	0,603
	11	pO2	0,733	0,600	0,444
	12	pCO2	0,567	0,585	0,683
	13	Bicarbonate	0,667	0,523	0,730
Mode	14	Pressure support	0,733	0,877	0,841
	15	Volume support	0,267	0,123	0,159
Disease probabilities			0.63	0.66	0.73

### Calculation of the ventilator settings with Artificial Neural Network

The second stage of the decision support system begins with the acceptance of the accuracy of the forms of entries. ANN used in the second stage of the system, is implemented via the MATLAB. Because of having the ability to learn complex nonlinear input-output relationships, use sequential training procedures, and adapt themselves to the data, we prefer to work with ANNs.

**Figure 3:**
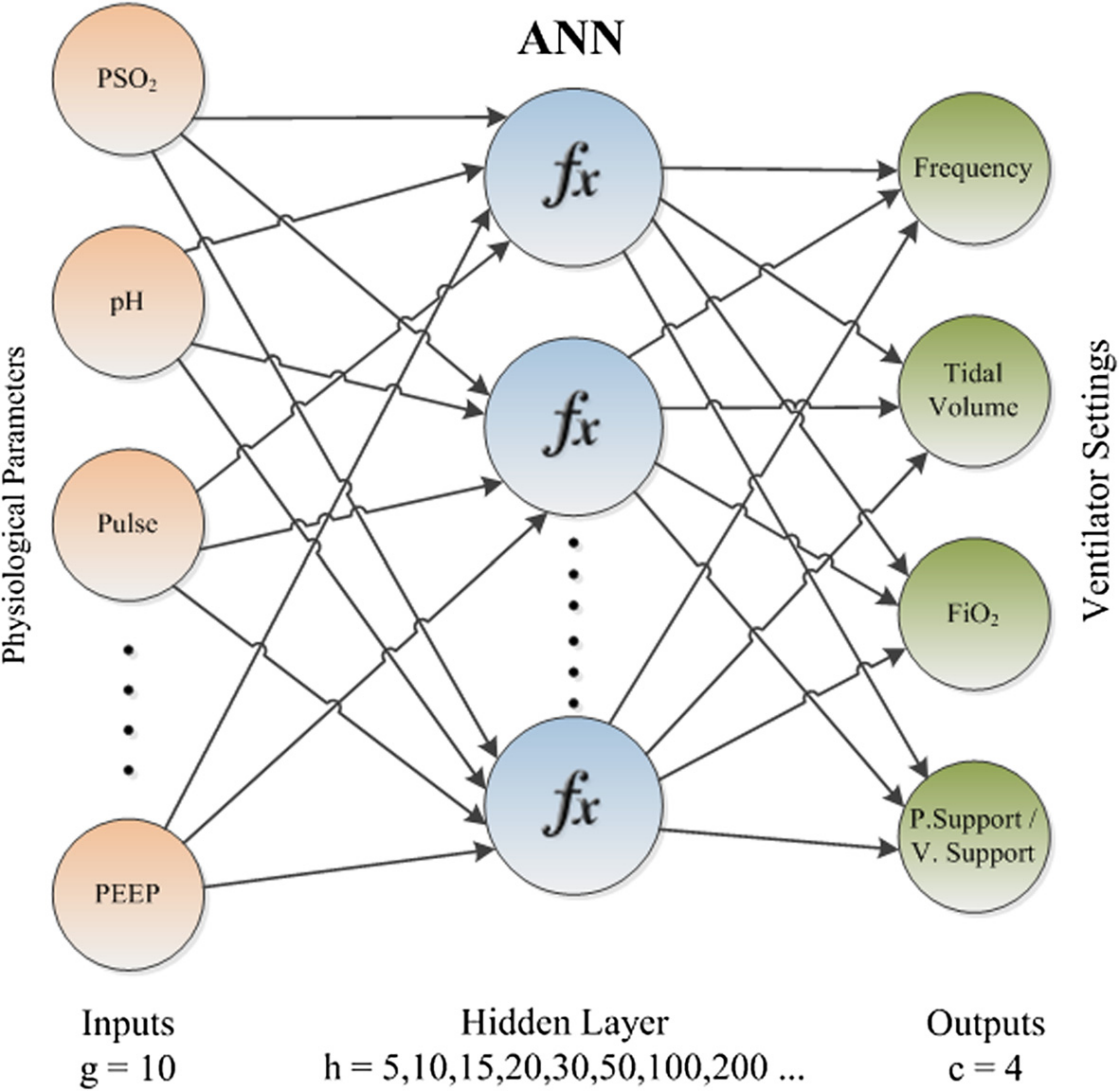
ANN architecture.

While 10 features are included in ANN as entries as the diagnosed disease, core body temperature, pulse, arterial systolic pressure, diastolic blood pressure, PEEP, *PSO*_2_, pH, *pCo*_2_ and bicarbonate, 4 ventilator settings are created: frequency, tidal volume, *FiO*_2_ and pressure support/volume support values. At the end of the second stage, values produced as a result are presented to the physician to be used in the treatment of the patient. Operational architecture of ANN we developed is presented in Figure 3. Input layer is the neurons which are holding the input values as patients’ data. Output layer is responsible to convey the produced ventilator settings -network data- to outside. Computations were performed in the hidden layers between these two layers. Each node can only take input from the previous layer of itself.

The function created for the modelling of ANN is given in (6). The initial weights multiplier values for 10 independent entries are randomly selected. At the end of the training, the optimum coefficients are found and the calculation is made.

(6) $$\begin{array}{lll} \text{ANN}\;=\; & i*\;(w_{1}*\text{SDisease}\;+\;w_{2}*\text{SCoreBodyTemp}\;+\;w_{3}*\text{SPulse}\; & \;\\ & +\;w_{4}\;*\;\text{SArterialSPres}\;+\;w_{5}\;*\;\text{SDiastolicBPres}\;+\;w_{6}\;*\;\text{SPEEP}\; & \;\\ & +w_{7}*\text{SPSO}2+w_{8}*\text{SpH}+w_{9}*\text{SPCO}2\; & \;\\ & +w_{10}*\text{SBicarbonate})\; \end{array}$$

ANN operates in two different modes. In order to calculate the frequency, tidal volume and *FiO*_2_ outputs, regression model has been used and the classification model has been used for the estimation of the pressure support/volume support outputs. For obtaining the highest performance in both models, different configurations have been tried. Various tests have been realized for the training method and the number of hidden layers [28], which are the factors affecting the performance of ANNs the most.

While working on the training method, 4 backpropagation and 2 supervised training algorithms have been used. The used backpropagation training algorithms are as follows: Levenberg-Marquardt, Bayesian Regulation, One step secant and BFGS quasi-Newton. For the other 2 supervised training algorithms, Cyclical order weight/bias and Sequential order weight/bias were preferred.

In order to find the number of hidden layers with which all the entire training algorithms give the best performance, layer numbers of 5, 10, 15, 20,30, 50 and 100 have been tried. Results for all tests are detailed in Section “Results and discussion”.

## Results and discussion

Prior to the second step of the system which is the estimation of the ventilator settings, the probabilities of the diseases were predicted and presented to the physician, and upon the verification of relevant disease or determination of a new disease by the physician, they were sent as parameters for the predicted model of ventilator settings.

Many tests have been made for the determination of the second stage parameters where the ventilator settings are proposed. We have applied k-fold cross validation model in our ANN experiments, and due to the size of the data, k is chosen as 10. In 10-fold cross validation each data set have been partitioned in ten folds, and iteratively nine of those are taken to train the ANN, so the last fold is taken for testing the learning of the network. In this way, 60 runs and their respective validations have been carried out, and the tests results are summarized in Table 2.

**Table 2:** Results for ANNs Using 10-fold cross validation

**Learning Alg./Output parameters**	**Mean** ***±*** **s**			
	**Frequency**	**Tidal volume**	**FiO2**	**PS/VS**
Bayesian regulation	90.474 ±0.956	88.733 ±0.867	89.608 ±1.002	93.644 ±0.756
One step secant	89.575 ±1.235	87.759 ±1.821	90.187 ±1.403	90.094 ±1.006
BFGS quasi-newton	82.563 ±0.997	75.775 ±1.945	76.626 ±1.874	82.113 ±1.187
Cyclical order weight/bias training	65.292 ±2.118	71.315 ±2.145	72.002 ±2.308	91.337 ±0.863
Sequential order weight/bias training	100 ±0	99.796 ±0.014	99.515 ±0.025	85.129 ±1.571

**Figure 4:**
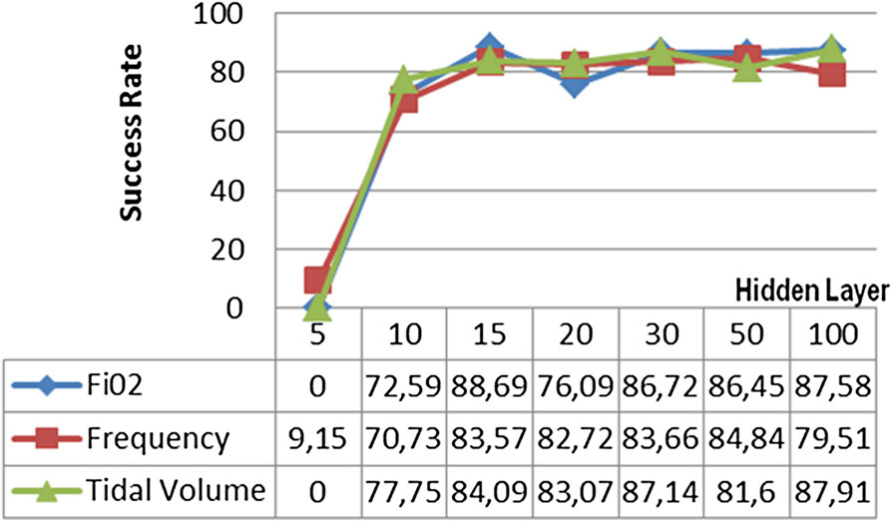
Levenberg-Marquardt backpropagation learning algorithm test results.

Firstly, Levenberg-Marquardt’s backpropagation learning algorithm has been tested by using different number of hidden layers in order to train the regression model through which *FiO*_2_, frequency and tidal volume parameters were proposed. The prediction rates obtained by using the Levenberg-Marquardt algorithm were calculated as 86,72% for *FiO*_2_, 83,66% for frequency and 87,17% for the tidal volume, when 30 hidden layers were used for the highest prediction percentage. Operation of the system from 15 hidden layers to 100 hidden layers without decreasing the accuracy below the average of 80,63% was completed in approximately 1.5 seconds. The graphic showing test results of the Levenberg-Marquardt algorithm is given in Figure 4.

**Figure 5:**
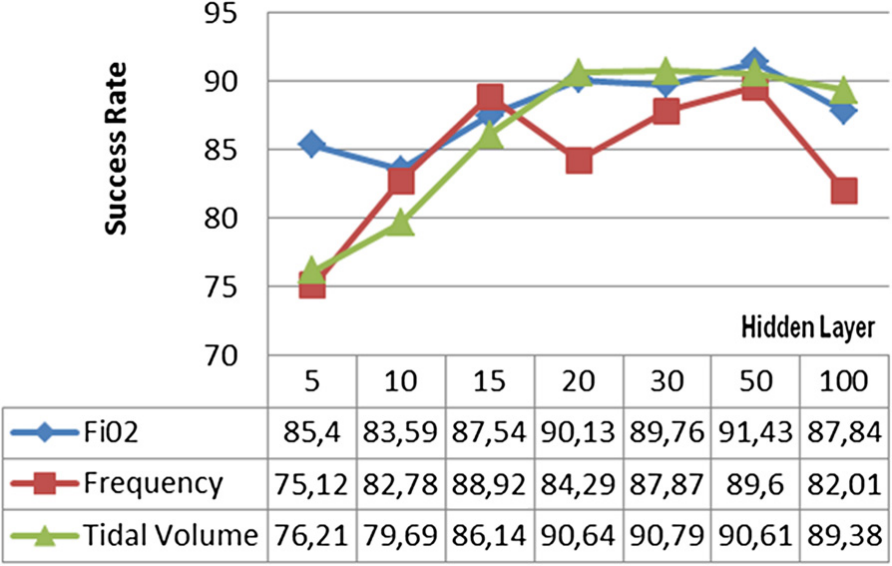
Bayesian regulation backpropagation learning algorithm test results.

Following the Levenberg-Marquardt algorithm, another backpropagation learning algorithm used in test was the Bayesian Regulation. In all the preferred hidden layer parameters used during the tests, the average success was never below 78,91%. The most successful test results were obtained by using 50 hidden layers following a process period of 2 minutes which were recorded as 91,43% for *FiO*_2_, 89,6% for the frequency and 90,61% for the tidal volume. When all the tests are examined in general, it is seen that the hidden layer parameter in the Bayesian Regulation backpropagation learning algorithm directly effects the time of operation. The time of operation which lasted for 2,7 seconds when 5 hidden layers were used increased to 637 seconds when 100 hidden layers were used. The success rates of the tests where the Bayesian Regulation algorithm was used are given in Figure 5.

**Figure 6:**
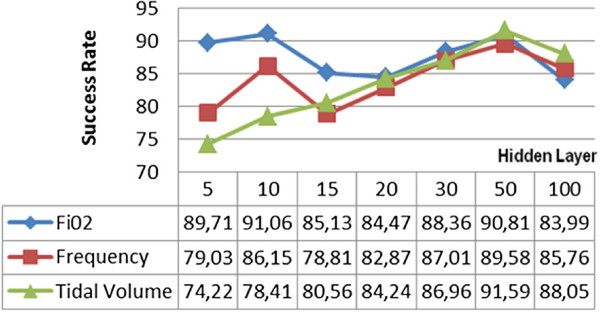
One step secant backpropagation learning algorithm test results.

Third backpropagation learning algorithm used in the tests is the One step secant. The operation time in the tests that used hidden layers from 5 to 100 as parameters has been completed within a range of 15 – 20 sec. The general average success rate was calculated as 80,99%, even for the test where 5 intermediate layers were used. In the test where 50 hidden layers were used and which had the highest success rate, the values for *FiO*_2_, frequency and tidal volume were 90,81%, 59,58% and 91,59%, respectively. A graphic showing the test result of One step secant algorithm is shown in Figure 6.

**Figure 7:**
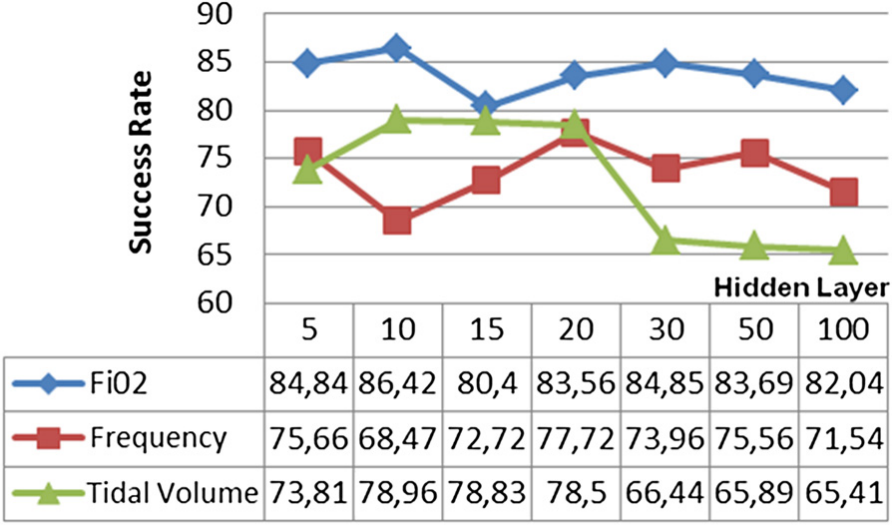
BFGS quasi-Newton backpropagation learning algorithms test results.

The test results where the BFGS quasi-Newton backpropagation learning algorithm was used gave the worst estimation percentages. Even in the best conditions, the success rates for *FiO*_2_, frequency and tidal volume were 83,56%, 77,72% and 78,5%, respectively. These percentages were obtained from ANN using 20 different hidden layers. Test results of the BFGS quasi-Newton algorithm are shown in Figure 7.

**Figure 8:**
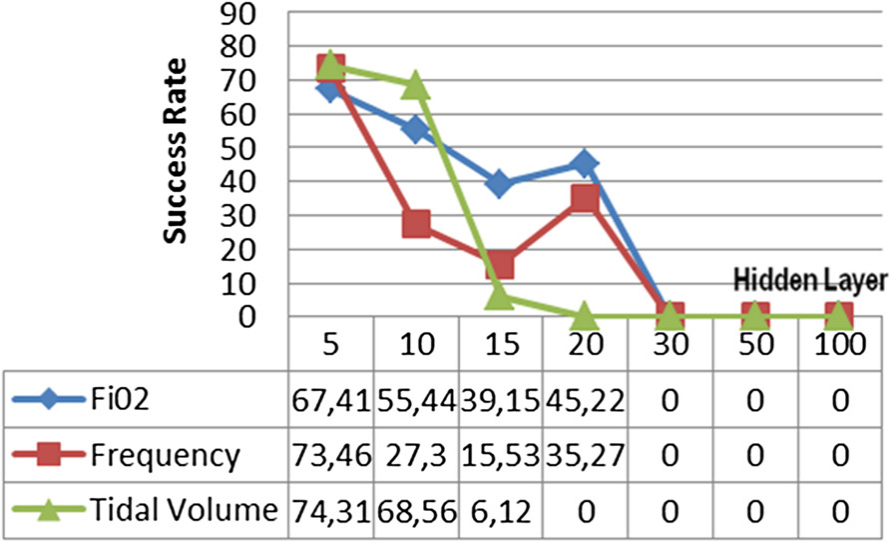
Cyclical order weight/bias supervised learning algorithm test results.

Two supervised training algorithms have been used as alternative to backpropagation learning algorithms we used for training ANN. The first algorithm is the Cyclical order weight/bias algorithm. No estimation was provided where higher numbers of hidden layers were used. Even in cases where a comparatively low number of hidden layers were used, the lowest success rate was obtained among all the methods. In ANN configuration with an operation time of 126 sec where 5 hidden layers were used, the estimation success rates for *FiO*_2_, frequency and tidal volume were 67,41%, 73,46% and 74,31%, respectively. The graphic regarding the tests conducted with all the Cyclical order weight/bias algorithms is shown in Figure 8.

**Figure 9:**
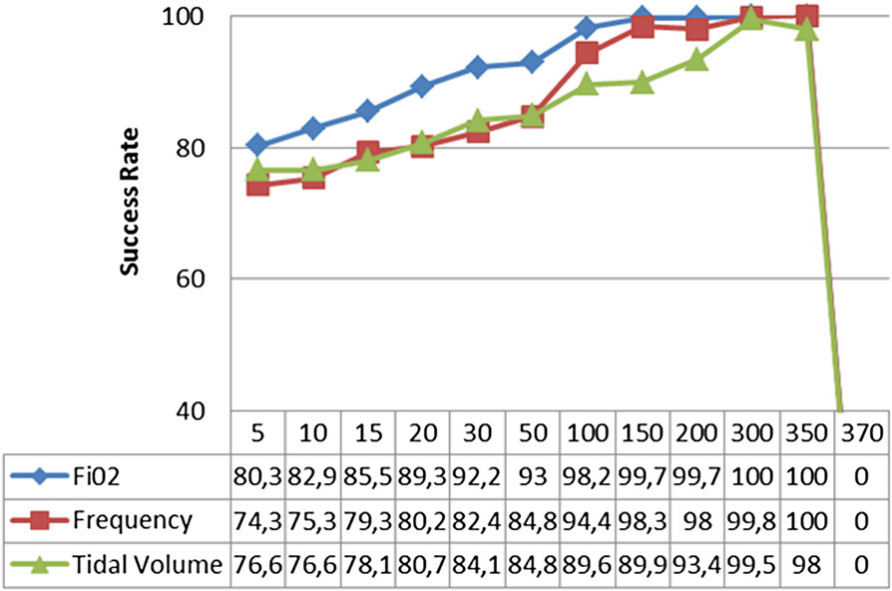
Sequential order weight/bias supervised learning algorithm test results.

Recent tests made in the regression model have been realized by using the Sequential order weight/bias supervised learning algorithm. It was a general observation that this algorithm was capable of accomplishing the operation at high speeds and in consistency with the highest number of hidden layers. This provided the highest success rate among all the tests using other algorithms. As per the configuration where 300 intermediate layers were preferred, the prediction success rates were 100% for *FiO*_2_, 99,81% for frequency and 99,54% for tidal volume, after an operation time of only 6 seconds. Tests made by using this algorithm are shown in Figure 9.

**Figure 10:**
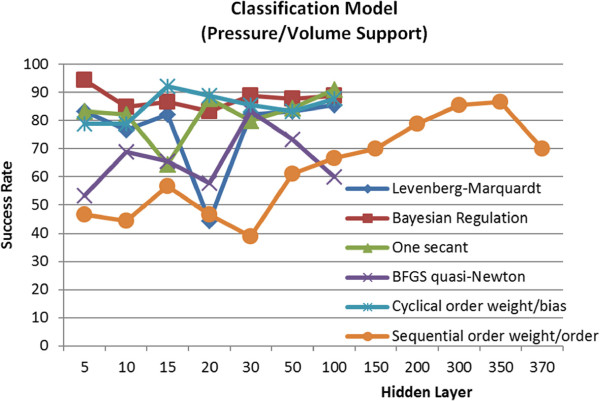
Test results of learning algorithms used in classification model.

In the tests for the classification of pressure support and volume support parameters, the highest success rates of 94,4% and 92,2% were obtained by the Bayesian Regulation backpropagation learning algorithm and the Cyclical order weight/bias supervised learning algorithm, respectively. When the operation time is considered as an important parameter, the Bayesian Regulation algorithm should be preferred in the first place due to having an operation time of 1.1 seconds. Values for the tests made for the classification model have been presented in Figure 10.

Among the tests we made for the regression and classification models, 300 hidden layer Sequential order weight/bias supervised learning algorithm was preferred for the prediction of *FiO*_2_, Frequency and Tidal Volume, whereas 5 hidden layered Bayesian Regulation backpropagation learning algorithm was preferred for Pressure Support/volume support parameter classification. Operation times and success rates of the hidden layer configuration that gave the most successful results are given in Table 3. During the tests, 6GB memory and Intel ^*®*^ Core ^TM^ i7-870 (8M Cache, 2.93 GHz) processor computers has been used.

**Table 3:** The most successful configurations and success rates provided by the training algorithms used in the regression and classification models

	**Regression model**						**Classification model**		
Learning algorithm	Hidden layer count	Process time (s)	FiO2	Frequency	Tidal volume	Average success	Hidden layer count	Process time (s)	Pressure support/ Volume support
Levenberg-Marquardtbackpropagation	30	1,4	86,72%	83,66%	87,14%	85,84%	100	37	85,60%
Bayesian regulationbackpropagation	50	121	91,43%	89,60%	90,61%	90,55%	5	1,1	94,40%
One step secantbackpropagation	50	17	90,81%	89,58%	91,59%	90,66%	100	25,1	91,10%
BFGS quasi-Newtonbackpropagation	20	12	83,56%	77,72%	78,50%	79,93%	30	29,6	83,30%
Cyclical order weight/biastraining	5	126	67,41%	73,46%	74,31%	71,73%	15	179	92,20%
Sequential order weight/bias training	300	6	100,00%	99,81%	99,54%	99,78%	350	12	86,70%

As seen from the examples provided in Table 3, when the results produced by the decision support system we proposed are compared to the ventilator settings actually used for obtaining data; it is shown that the success rates in the prediction of frequency parameter, tidal volume parameters, *FiO*_2_ parameters and pressure support/volume support parameter are 99,81%, 99,54%, 100% and 94,40%, respectively.

Furthermore, proposed models field tests conducted on 14 patients of 2 different doctors show that, the system trained with 158 patient data recommended the ventilator settings with 95,3% accuracy. This percentage has remained low compared to the simulation results, but this ratio provides a satisfactory grade for the use of our proposed system in real life. It is clear that, gaining more data to train the system increase this success ratio.

## Conclusions

This article has aimed at making the parameter selection process in the ventilator treatment period of the respiratory tract patients independent from the physician, via the decision support system proposed. As seen from the experimental test results, the system can propose the frequency, tidal volume, *FiO*_2_ and pressure support/volume support parameters by an accuracy rate of 98,44% on simulations and an accuracy rate of 95,3% on field tests. The incorrect suggestions has small deviance to optimum values, which are harmless to patients in case of application.

The training of the ANN in the system has been realized with the physiological data from 158 patients. The rate of accuracy in the system’s prediction capability will increase with the use of data from higher number of patients. Thus, non-physician operators will also be able to use the system towards the determination of the ventilator settings in case of emergencies.

Improvement of the project as an automation system can be provided by the transmission of the physiological values of the patients to our decision support system from the hospital database and the direct installment of the treatment parameters preferred by the system in the ventilator device, without a mediator. For further studies we are going to identify the parameters which have less impact on results. Elimination of these parameters will speed-up the execution periods.

## Competing interests

The authors declare that they have no competing interests.

## Authors’ contributions

FPA conceived of the study and participated in its interpretation, analysis, development, testing and writing of the manuscript. EA was responsible for the acquisition and interpretation of data. AA contributed to the evaluation of the results and preparation of the manuscript. BSY organized the study and contributed to the reviewing of the manuscript. All authors read and approved the final manuscript.

## Pre-publication history

The pre-publication history for this paper can be accessed here:

http://www.biomedcentral.com/1472-6947/14/3/prepub
